# Quantitative assessments of honeybee colony’s response to an artificial vibrational pulse resulting in non-invasive measurements of colony’s overall mobility and restfulness

**DOI:** 10.1038/s41598-024-54107-8

**Published:** 2024-02-15

**Authors:** Martin Bencsik, Adam McVeigh, David Claeys Bouuaert, Nuno Capela, Frederick Penny, Michael Ian Newton, José Paulo Sousa, Dirk C. de Graaf

**Affiliations:** 1 Nottingham Trent University, Nottingham, UK; 2 Harry Butler Institute, Murdoch University, Perth, Australia; 3 Honeybee Valley, Ghent University, 9000 Ghent, Belgium; 4 Centre for Functional Ecology, Department of Life Sciences, Associated Laboratory TERRA, University of Coimbra, Coimbra, Portugal

**Keywords:** Biomedical engineering, Acoustics

## Abstract

In this work we aim to provide a quantitative method allowing the probing of the physiological status of honeybee colonies by providing them with a gentle, short, external artificial vibrational shockwave, and recording their response. The knock is provided by an external electromagnetic shaker attached to the outer wall of a hive, driven by a computer with a 0.1 s long, monochromatic vibration at 340Hz set to an amplitude that occasionally yields a mild response from the bees, recorded by an accelerometer placed in the middle of the central frame of the colony. To avoid habituation, the stimulus is supplied at randomised times, approximately every hour. The method is pioneered with a pilot study on a single colony hosted indoors, then extended onto eight outdoors colonies. The results show that we can quantitatively sense the colony’s overall mobility, independently from another physiological aspect, which is phenomenologically explored. Using this, a colony that is queenless is easily discriminated from the others.

## Introduction

European honeybee colonies establish themselves in natural and man-made dark cavities, and cannot usually be seen unless the beekeeper inspects the hive by opening it invasively. Although the traffic of bees at the entrance of the hive gives an indication of its status, in the wintertime all foraging can cease for weeks or even months, and it is not even possible to tell whether the colony is alive or dead. Nowadays a thermal camera can be used to distinguish alive, warmer colonies from dead ones, but in the past many beekeepers have been simply knocking on their hives, with their hand, to check and listen for a positive buzzing response, indicating the liveliness of the colony.

The potential relevance of this simple test has never been investigated properly, even though it perhaps holds a key to non-invasively assess at least one aspect of the overall physiological status of the colony. Indeed, knocking on the hive, in order to then assess the detected response can be seen as exploiting one of the super-organisms’ reflex arc.

Usually undertaken on vertebrates, the reflex arc test requires a stimulus to be provided to the living organism, resulting in a “response” that can be measured quantitatively. As the response is achieved through a neural pathway (which may or may not require the brain), its features (i.e. magnitude, time duration, etc..) often yield an assessment of an aspect of the status of the organism’s central nervous system, reflecting a particular detail of its physiological status. Since the reflex arc is involuntary, the assessment is free from subjective bias. Such tests must however not be undertaken at extremely regular times or in too close succession, in order to avoid habituation in the animal under investigation.

Some reflex arc tests have proven to be extremely useful, particularly when simplicity is combined with the relevance of the assessment. In new-born humans, for instance, the plantar reflex^1^ and the Moro reflex^2^ are used daily all over the world for the early detection of spasticity.

Although knocking on a honeybee hive is well known to indicate whether live bees reside in the box or not, one author^3^ also suggested that the buzzing response should be termed the ‘hiss’, and that its decay length and magnitude reveals the presence of brood in the hive. We also suggested^4^ that the honeybee whooping signal can be stimulated by providing a vibrational knock to a hive, and that it could be the result of a reflex arc which holds further potential to assess the colony status.

When continuously applying narrow-band vibrational wave forms, it has been shown^5,6^ that honeybees can be immobilised for time-durations as long as four hours, and that their mobility is restored as soon as the stimulus is stopped, with no apparent secondary effects. When applying a random, incoherent, broad-band, long-lived ‘white noise’ vibration, immobilisation of honeybee has also been demonstrated^7^. A sharp knock delivered onto a hive corresponds to a coherent broad-band, short-lived wave form, therefore it may also cause a brief immobilisation of the colony, immediately prior to the ‘hiss’ response. Honeybees also become immobilised under vibrations that originate from within the external natural world (there might be vibrations induced by thunder, or the tree they reside in might be knocked by branches) and from within the colony itself. Many beekeepers will have inspected a frame, in the swarming season, on which a virgin queen is in the process of providing a ‘tooting signal’, resulting in the spectacular sight of worker bees becoming immobilised on the entire frame, in perfect synchrony with the queen’s vibrational signal. This has led one author^8^ to suggest that the freezing response aids the queen to communicate with the colony, as all workers on the frame remain ‘vibrationally silent’.

In our work we delivered, for several months, an automated, highly repeatable, short-lived, weak vibrational pulse to a collection of nine hives, at randomised times approximately one hour apart. This resulted in a mild reaction of the colony, which was recorded by means of an accelerometer residing in the middle of the central frame of each hive^4,9^. We show that the measured response comprises of at least two independent sections, a short-lived decrease of honeybee vibration originating from their immobilisation, followed by a relatively long-lived increase of the signal with an exponential decay. We demonstrate that the first part reveals the colony’s overall mobility, and we suggest that the second part correlates with the colony’s restfulness, or lack of stress.

## Results

**Figure 1:**
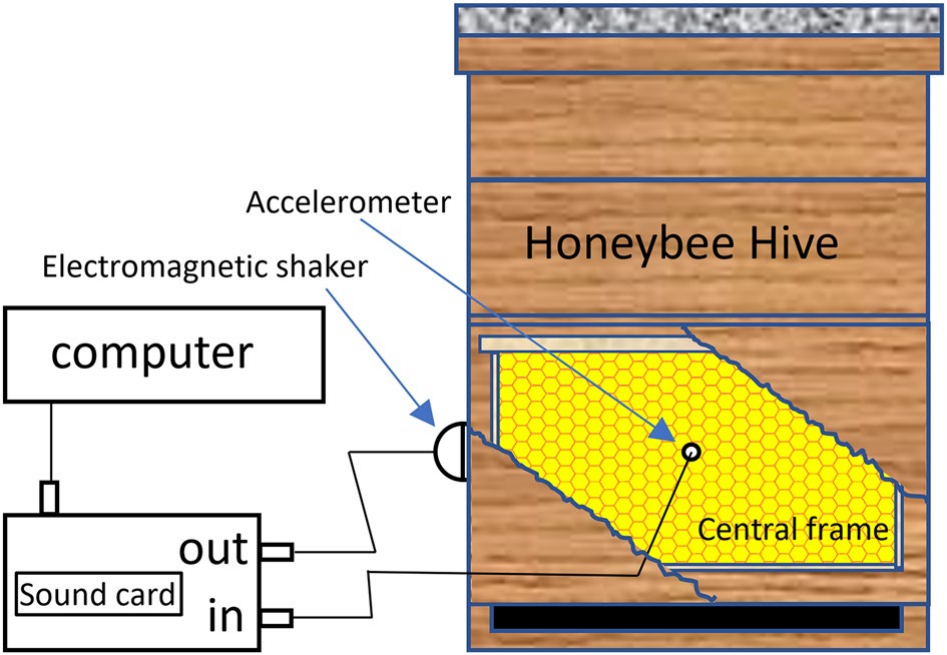
Sketch of the experimental set up. The front face of the hive has been cut out, to allow the visualisation of an inner frame with the accelerometer.

**Figure 2:**
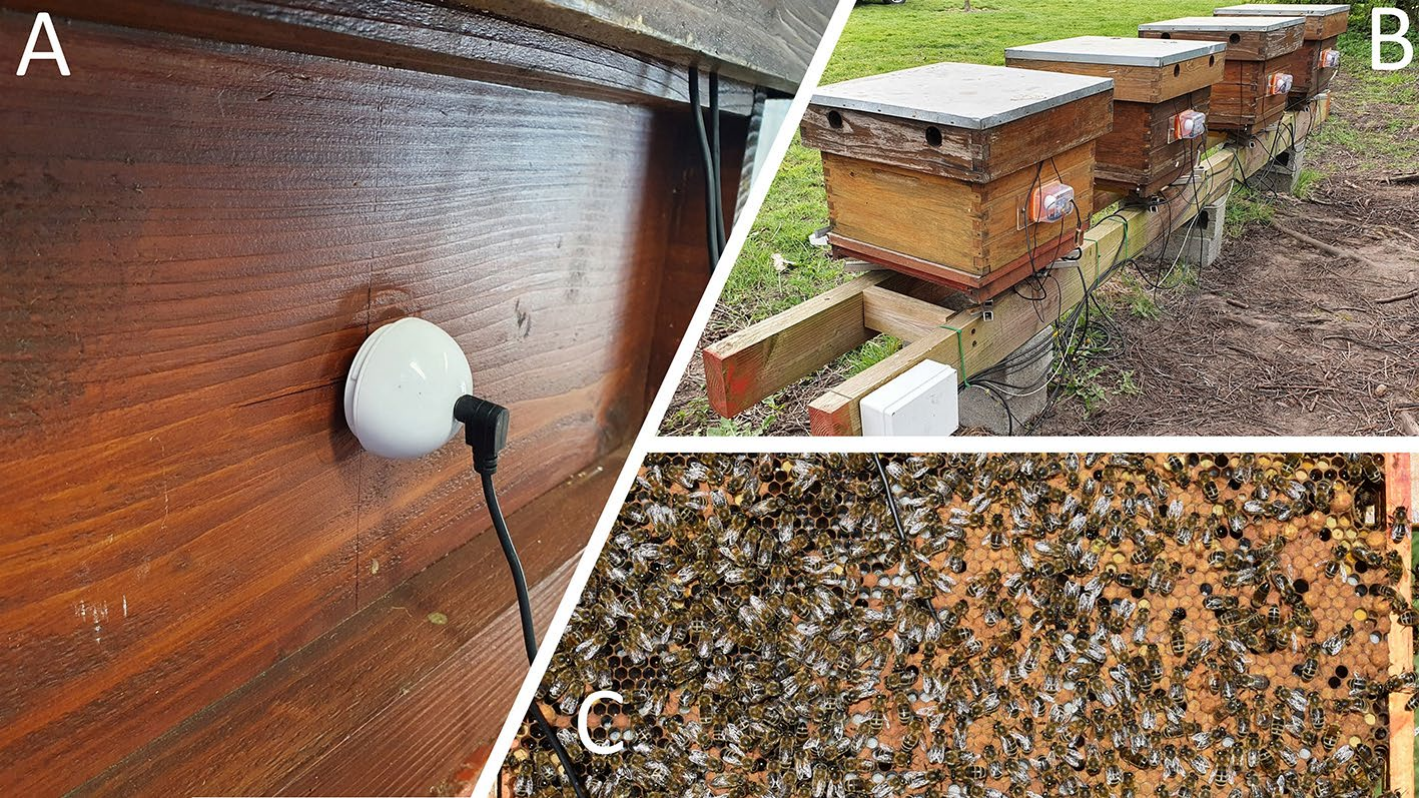
(**A**) Photo of the electromagnetic shaker (without protective box) secured to the outer wall of the brood box of the indoor hive, for the pilot study. (**B**) photo of a collection of four honeybee hives in Gent, Belgium, equipped with the same electromagnetic shakers, in individual weatherproof boxes. (**C**) photo of one side of the central frame of a honeybee hive equipped with an accelerometer inserted in the middle of the honeycomb. Although the black wire reveals the presence and the location of the accelerometer, the sensor itself is not visible anymore, as bees have built cells around it and are using them normally for brood, honey and bee-bread storage.

In order to provide an automated, repeatable vibrational knock onto the hive, we ended up using a low output power (1W) electromagnetic shaker (‘Mighty Boom Ball’, Focus Multimedia Limited, Rugeley, UK) housed, together with its pre-amplifier, in a weatherproof box, tightly secured to the outer wall of the hives. The colony’s reaction was recorded by means of an accelerometer (8 hives equipped with 805M1-0020, TE Connectivity, CH and one ‘pilot study’ hive with 4507B002, Brüel and Kjær, DK) placed in the honeycomb in the middle of the central frame of the hive as previously reported^4,9^, see Figs. 1 and 2.

## Pilot study

Using the ultra-sensitive accelerometer (1V/g) on a hive, kept indoors, for the pilot study, we failed to detect any reaction from the bees when using a single voltage step change, whatever its magnitude allowed by our sound card, when trying to mimic an electronic ‘knock’. Obtaining a substantial physical ‘knock’, with this particular transducer, would require a voltage step much higher than we could deliver. However, when exploring the use of a ‘beep’ waveform, a very short pulsed monochromatic wave (a pure sinusoidal wave shaped by a gaussian envelope with a full width half maximum of 30 ms), by gradually increasing its frequency, ‘whooping signals’ were heard from 300 Hz onwards, typically 200 ms after the pulse, with enough repeatability to convince us that a very mild reaction was caused by our artificial stimulus. Based on this initial promising reaction, we ran all further long-term studies in this work using a ‘beep’ pulse centred at 340Hz (and we also show that this initial choice was not the optimum frequency). By knocking with the hand, extremely gently, onto the hive, a reaction could be heard from the colony similar to that obtained when using our artificial pulse.

**Figure 3:**
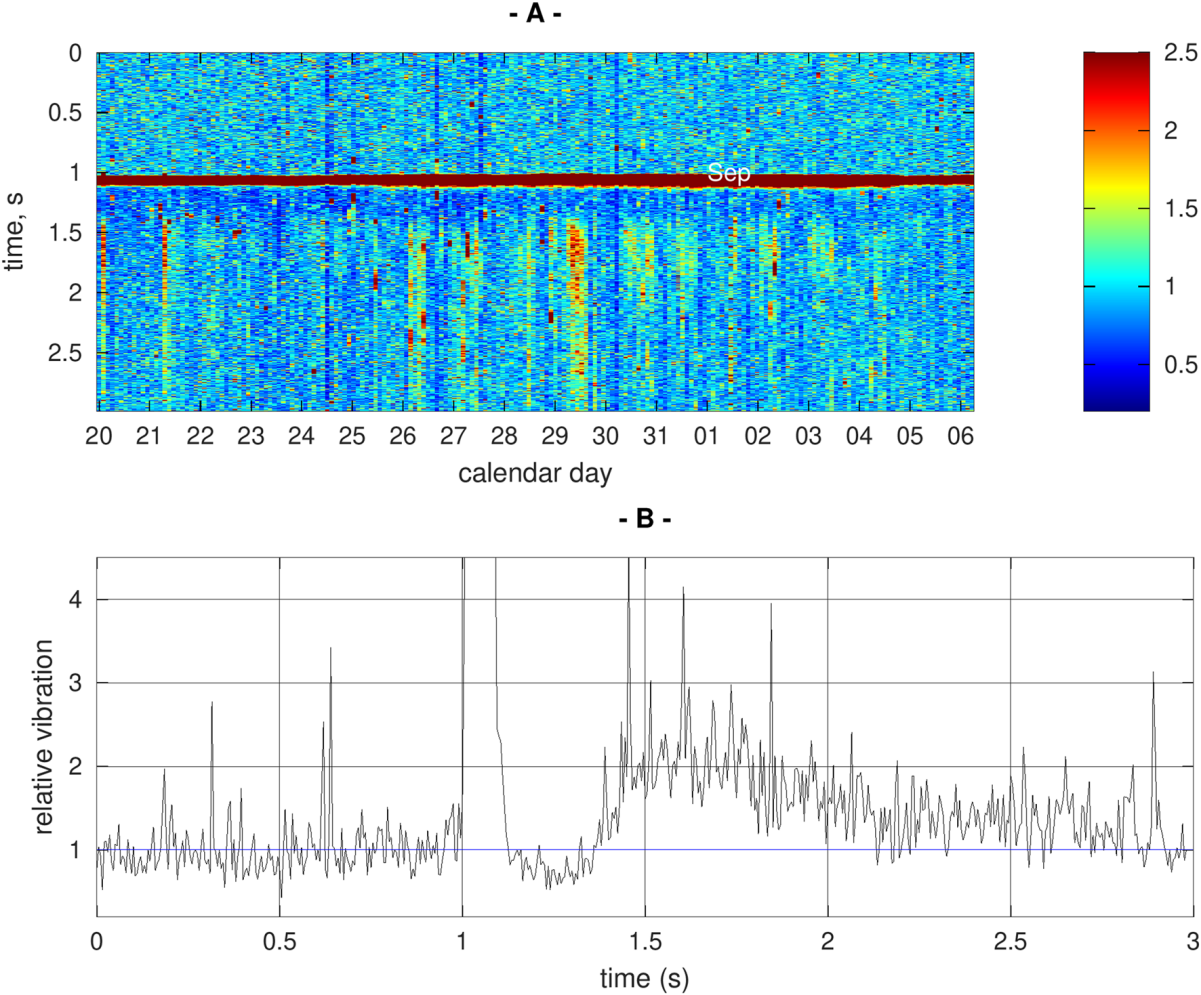
(**A**) A collection of three-second-long accelerometer recordings, in late August and early September 2021, with the time of the recording on the horizontal axis, the colour of the pixel intensity conveying the vibrational magnitude relative to that found before the stimulus, and the time relative to the artificial stimulus on the vertical axis. The colour coding has been adjusted to optimise the visualisation of the bees reaction, causing a clipping of the much stronger signal recorded during the application of the short pulse (red horizontal line), which is taking place exactly one second after the start of each recording extract. (**B**) A plot of a particular recording taking place on the 29th Aug 2021 with a remarkably strong colony response, which is also made available to the reader in Audio S1.

We proceeded to wire up the shaker to a computer sound card, and we wrote software to automatically send the pulse at randomised times, approximately one hour apart, whilst the accelerometer signal recording the vibrations originating from the bees was continuously recorded on a large external hard disk, as done in our previous works^4,9^. After a few days of running the automated experiment, the data was inspected, see Fig. 3. We were very excited to see evidence of a consistent, brief, mild (around 20%) vibrational signal decrease immediately after the pulse, followed by a less consistent, enhanced vibrational trace that is much longer lived and intense. Furthermore, we saw clear temporal patterns in the positive response of the bees, evolving relatively slowly with time, indicative of physiological changes taking place in the colony. These appear independent from the slow evolution of the short-lived vibrational loss, suggesting that both features originate from two independent honeybee colony physiological features.

Following the application of the pulse, whooping signals were often recorded, but by critical listening they appeared to come from a small collection of a few isolated individuals, whilst the buzzing enhancement with the exponential decay demonstrated in Fig. 3 clearly comes from a very large collection of honeybee individuals, and is therefore a much more relevant signature reflecting the colony. An accelerometer recording taking place on the 29th Aug 2021, with a particularly large buzzing response, is showcased in Fig. 3B, and is made available to the reader as Audio S1. The short-lived signal decrease cannot be perceived by critical listening, whilst the buzzing of the bees induced by the pulse can clearly be heard.

We now proceed to carefully quantitatively analyse both the short-lived signal decrease and the long-lived signal enhancement.

### Signal decrease and bees overall mobility

This indoor hive has already been described elsewhere^10^, and also comprises of a transparent stage, above it, into which the frame with the accelerometers can be temporarily lifted, in order to simultaneously record videos of the bees and the corresponding accelerometer signals. With the frame lifted in the observation transparent box, we filmed the bees residing on the honeycomb whilst driving a succession of ‘beep’ pulses (every 2.2 s) with the same electromagnetic shaker, secured directly on a metal rod holding the frame above the colony. Said ‘beep’ pulses had exactly the same temporal characteristics as the ones described before, but the sinusoidal wave’s frequency was changed from 0 to 2000 Hz in steps of 50 Hz. The relative mobility of the bees on the frame was quantitated, without absolute calibration, by calculating the mean of the (grey-scaled) difference image between any two consecutive frames. As the bees slow down and become immobilised, this quantity decreases, and in theory reaches zero when all the bees are totally immobile. This allows us to correlate in time, within one 50th of a second, the evolution of the (visually assessed) bees’ relative mobility with the (accelerometer assessed) application of the vibrational pulses. The full experiment is supplied to the reader as a video (Supplementary Video 1).

**Figure 4:**
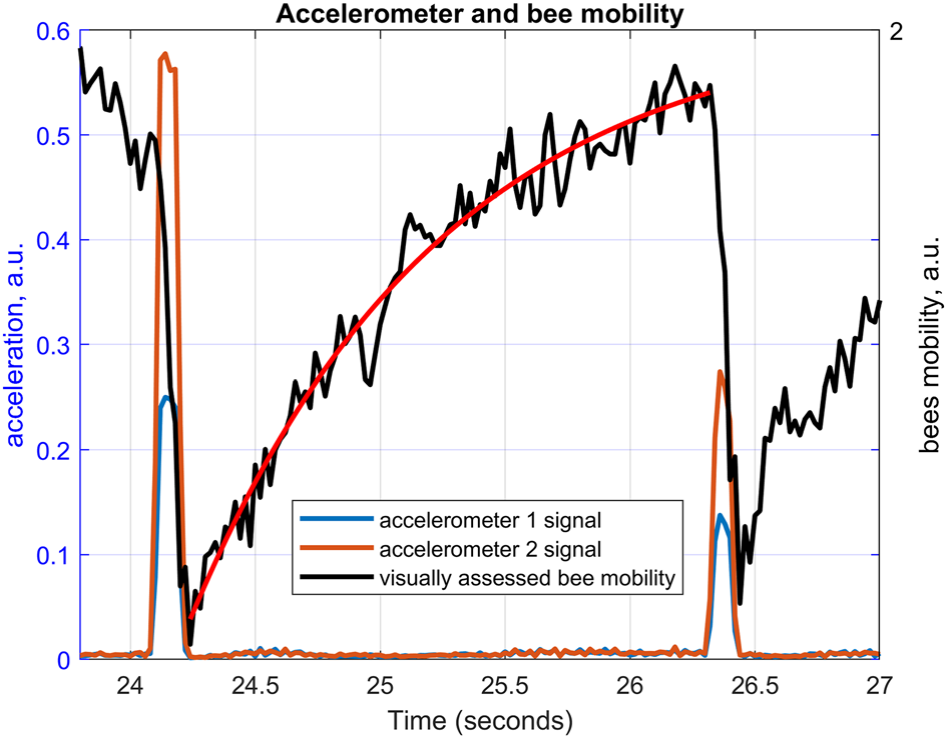
Comparison of the time course of the honeybees relative mobility (black curve), assessed by video analysis, with that of the vibration driven into the honeycomb (blue and orange curves) for two pulses respectively driven at 500 Hz (left) and 550 Hz (right). The red curve is a best-fit exponential recovery function with a time constant of one second. The bees immobilisation is maximised at the end of the application of each pulse. The frame had two accelerometers, 7 cm apart, on a horizontal line in the middle of the honeycomb, the difference between the signals demonstrates the local variations of the magnitude of the vibration reaching different parts of the honeycomb.

The results (Fig. 4) show that the short pulse is indeed immobilising the bees, that the immobilisation is maximised immediately at the end of the application of the pulse, that the effectiveness of the process is maximised with a pulse centred on 500 Hz, and that the bees recover their original mobility with an exponential recovery with a time constant that varies depending on the applied pulse’s frequency (the time constant is 1 s for the pulse centred at 500 Hz, and the Signal to Noise Ratio of the curve representing the bees mobility is insufficient at most other frequencies to allow the assessment of the corresponding time constant).

**Figure 5:**
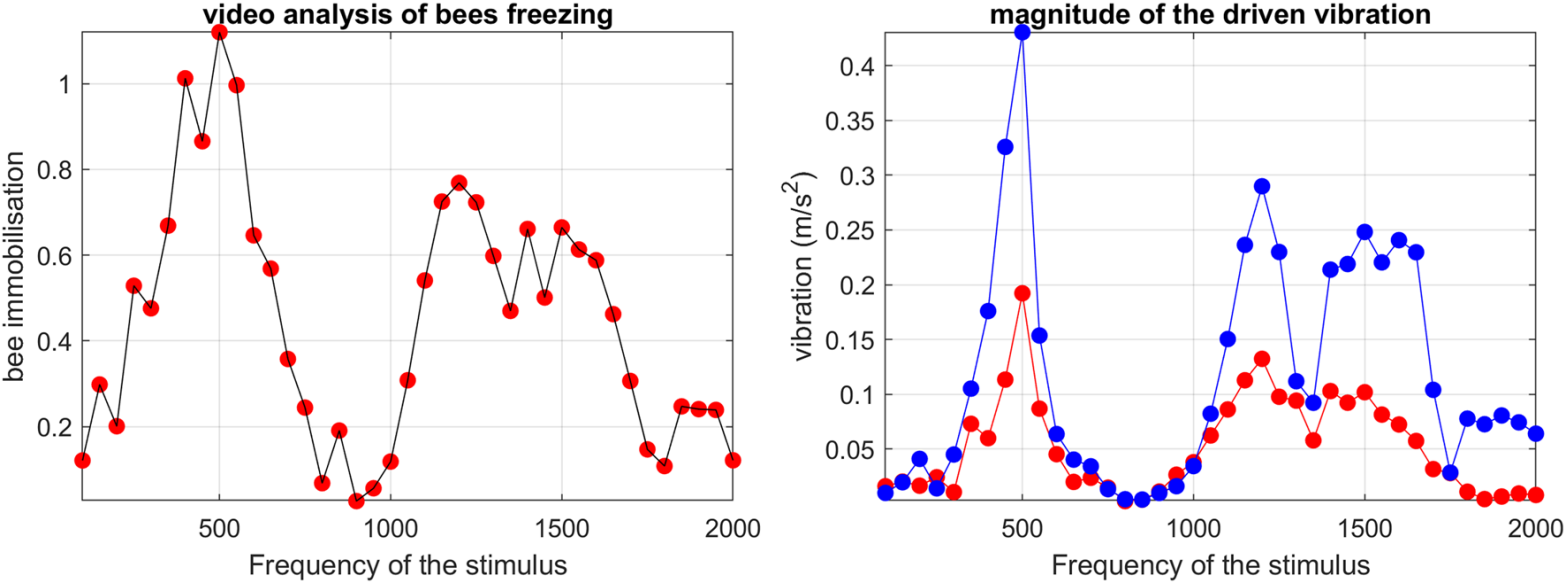
Left—Variations of the relative immobilisation of the bees obtained with a short pulse centred on a frequency varying between 50 and 2000 Hz. The vertical axis is the magnitude of the depth of the trough shown in Fig. 4. A clear maximum is reached around 500 Hz. Right—Variations of the magnitude of the vibration in the honeycomb at two different locations, due to the application of the pulses, at different pulse frequencies. The two curves are similar, and also resemble the one on the left, indicating that their variations are dominated by the frequency response of the electromagnetic shaker transducer. Coincidentally, the immobilisation phenomenon’s most sensitive frequency is thought to be around 400 Hz^8^ allowing an excellent use of our inexpensive transducer, at least in terms of optimising the measurement of the bees mobility.

We used the value of the curve representing the bees mobility found immediately after each pulse to assess the effectiveness of the immobilisation as a function of the applied pulse’s frequency (Fig. 5). The data closely follows the trend seen for the magnitude of the vibration perceived at the honeycomb, as measured by the two accelerometers embedded in the honeycomb, and therefore mostly reflects a combination of (i) the frequency dependence of the electromagnetic shaker’s output, and (ii) the vibrational modes of the honeycomb under investigation. The effectiveness of immobilising bees as a function of the driven frequency has been carefully assessed elsewhere^8^ and reaches a maximum around 400 Hz.

### Signal increase following the pulse

**Figure 6:**
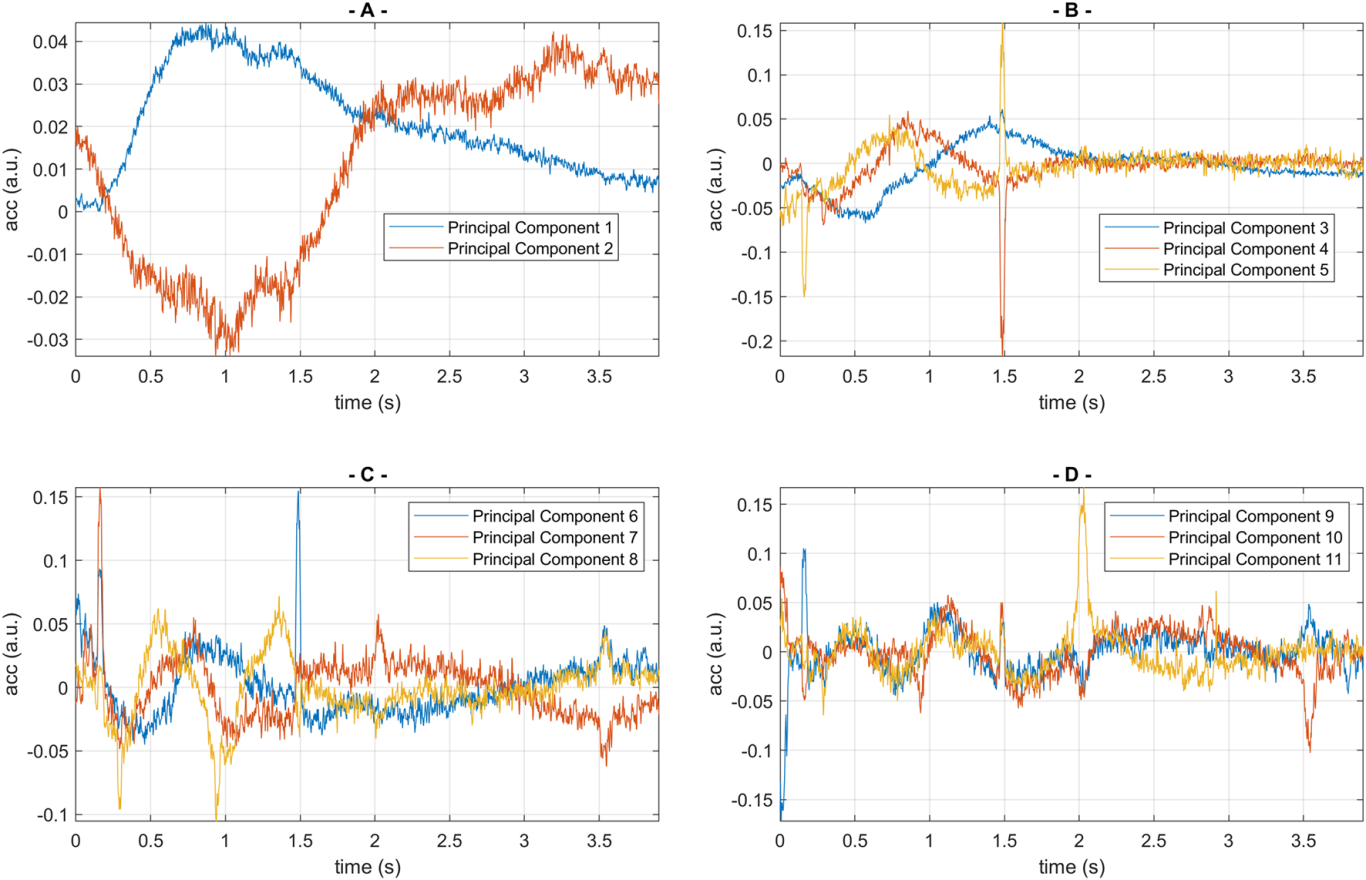
(**A**) PC components of rank 1 and 2, following a PCA search on the collection of accelerometer signals in the 4 s following the application of the pulse (See Fig. 7). The curves reflect the two main features of the slowly varying time-course of the positive collective buzzing response, and are totally free from short, pulsed artefacts. (**B**–**D**) PC components of increasing rank, from 3 until 11. Although some features of the positive slowly varying buzzing response can also be seen here, the curves are increasingly dominated by the envelope of the whooping signals stimulated after the artificial vibrational pulse.

**Figure 7:**
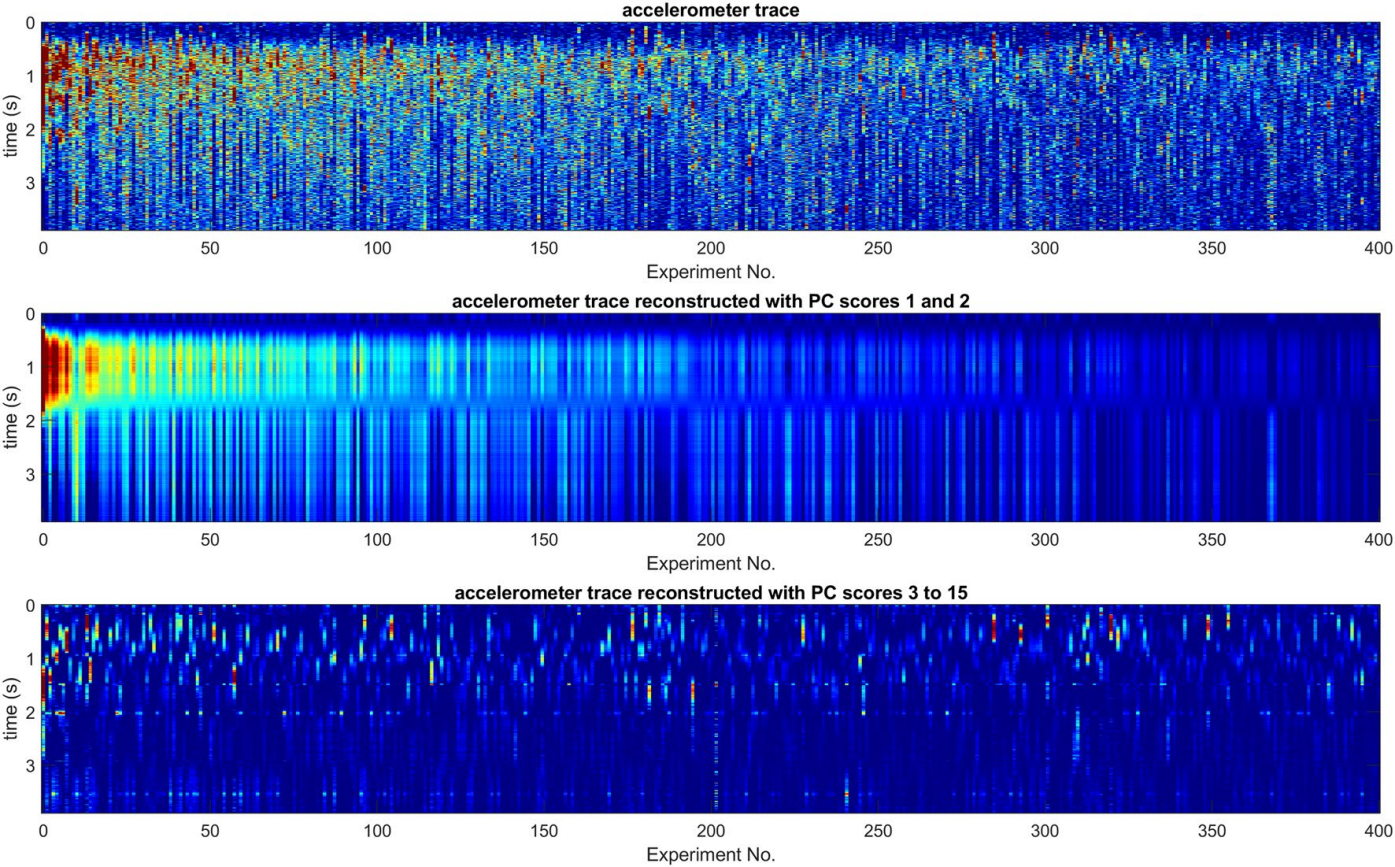
Top—Vibrational signals collected in the four seconds following the application of the stimulus, ordered according to their first PC score, with the strongest pulse on the left. Only the 400 strongest signals are showcased. The colour of the pixel conveys the vibrational magnitude (in arbitrary units), after the mean of the signal before the stimulus has been subtracted. Middle—Same signals as above, reconstructed by using only the first two PC scores, revealing with improved clarity the slowly varying buzzing response, which appears to peak around 1 s, comprising of independent short lived and a long lived contributions. Bottom—Same signals as in the top, reconstructed by using PC components with ranks between 3 and 15, revealing the presence of whooping signals, mostly in the 2 s following the application of the artificial pulse.

Due to our strategy where the smallest possible stimulus was driven, just about capable of causing a reaction, the signal changes showcased in Fig. 3 are low, compared with the environmental vibrational noise, but we have thousands of experiments to explore, in order to better characterise variations of interest. In our pilot experiment, between its start (20th Aug 2021) and its end (10th Dec 2022), 5595 pulsed stimulations were applied to the colony at randomised times, approximately an hour apart. The oversampled vibrational signals collected after the pulse were smoothed by using a moving average over a window of 4.5 ms, then fed to a PCA (Principal Component Analysis) search, in order to identify the largest deviations residing in the collection of measurements. The individual measurements were then sorted in order of similarity, by using the decreasing value of the first PC score, and stacked from left to right to allow a clearer visualisation of the variations seen in the positive vibrational response of the colony, see Figs. 6 and 7. The results (Fig. 6) show that the presence of whooping signals inherently become confined to the PC components of ranks greater than three, allowing a nearly perfect automatic discrimination of the slowly varying, collective buzzing response and the short lived whooping signals originating from a few individuals. Using only PC components of rank 1 and 2 (Fig. 7), the typical trace of the buzzing response can be examined with a clarity vastly improved compared with the raw data. Using a limited collection of PC components greater than three, the whooping signals (and other signals originating from a few individuals near the accelerometer) are seen to all take place within the first two seconds of the data following the application of the artificial pulse. Within the positive response of the bees reacting to the applied pulse, there is no obvious correlation between the strength of the buzzing response and the timings or density of the whooping signals. The reader is supplied with the fifty loudest envelopes showcased in Fig. 7, individually, together with the original accelerometer track, to allow their critical listening to validate the claim that we have automatically discriminated the buzzing response from the localised whooping signals (Supplementary Video 2).

### Vibrational signal perceived by the bees

**Figure 8:**
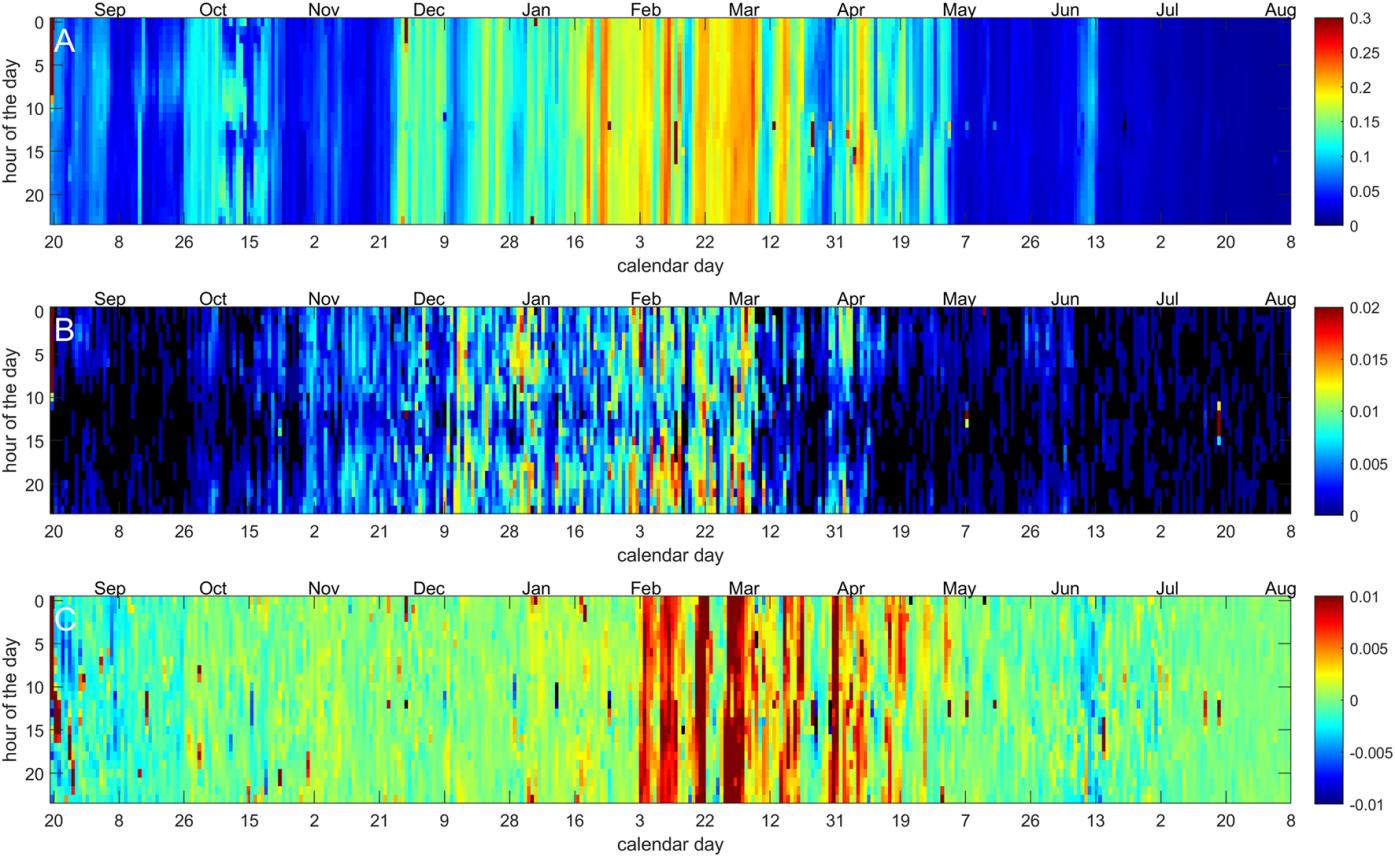
(**A**) Variation of the magnitude of the vibration reaching the honeycomb on which the bees response is measured. The vertical axis is the time of the day, the horizontal axis the day of the year, data has been interpolated in order to allow hourly visualisation (in reality pulses are driven at randomised times, approximately hourly) and pixel intensity reflects the magnitude of the vibration on a linear scale, in arbitrary units. (**B**) Variation of the magnitude of the positive response of the bees within the four seconds that follow the artificial pulse, with the same formatting as seen in -A-. The pixel intensity reflects the subtraction of the mean vibration recorded after the pulse, from the mean vibration recorded one second before the pulse. (**C**) Variation of the magnitude of the negative response of the bees within the 0.25 s that follow the artificial pulse, with the same formatting as seen in -A-. The pixel intensity reflects the subtraction of the mean vibration recorded 0.25 s after the pulse, from the mean vibration recorded one second before the pulse.

Although we are driving the exact same vibrational signal at the hive wall each time we run an experiment, the waveform has to propagate through a complex and dynamic structure, the hive itself, before it is eventually perceived by the bees residing on the frame. We checked the repeatability of the vibration that reaches the bees, simply by looking at the magnitude of the pulse detected by our accelerometers (Fig. 8A). Surprisingly, the vibration reaching the bees is seen to vary substantially, in amplitude, by up to one order of magnitude across the year. It is at its highest in March when the frame, and the colony, is very short of resources, and at specific times of the year, it can also vary considerably within one day. In spite of this, we seem to have been driving a signal that satisfies the threshold required for stimulating the bees’ positive reaction (Fig. 8B): in mid-December, for instance, a relatively weak vibrational strength generates a very large bees response, whilst a stronger signal reaching the bees in October has hardly any effect at all. Our ability to have successfully probed the bees mobility, all year round, is however less convincing (Fig. 8C): substantial bee mobility is only successfully measured in August, September and June, strongly suggesting that most of the time we failed at delivering a strong enough vibration for the exploitation of this particular phenomenon. At times (in the winter) when the colony’s mobility is expected to be near zero and where the positive buzzing response is high, note that even the 0.25 s of signal following the pulse demonstrates a vibrational enhancement (see February and March).

### Multiple hives study: phenomenological approach to the bees positive reaction

The pilot study suggests that the positive reaction is hardly measurable, at all, during the active season, becomes extremely high when the colony clusters for the winter, and is always reduced in the middle of the day. We proceeded to repeat the experiment on a collection of eight honeybee hives kept in the same apiary, in Nottinghamshire, UK, in order to start exploring the generalisation of some of our results. The same experiment was translated on these, but ten times less sensitive accelerometers were used (805M1-0020, TE Connectivity, CH), and the electromagnetic shakers were housed in a weather proof box. All eight colonies were stimulated at randomised times, approximately every hour, but all at exactly the same times, in order to avoid the possibility of a colony being artificially stimulated by a neighbour one. For the purposes of the ‘B-GOOD’ EU funded project, every colony was also manually carefully inspected every three weeks, allowing us to have an excellent record of the subjectively assessed status of each hive. One of the eight colonies (Colony No 3) went through the active season with great difficulties (whilst the seven others did not exhibit any major health challenges). These bees lost their queen in the spring, they were artificially ‘requeened’ and started to improve for two weeks, after which they then lost their second queen and deteriorated across the summer, all the way towards failure, until it was replaced with a fresh colony on September 21st.

**Figure 9:**
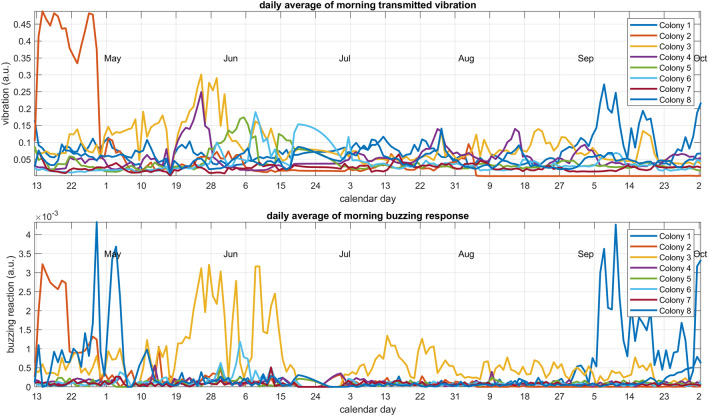
TOP—Daily average undertaken on the morning data only, of the magnitude of the artificial pulse reaching the bees, for 8 colonies residing on the same apiary. Large variations are seen, but the vibrations reaching colony No. 3 are not particularly high. BOTTOM—Daily average undertaken on the morning data only, of the positive, buzzing response that follows the artificial pulse, for the same 8 colonies. The response of the queenless colony No. 3 is substantially higher than that of any other colony, and comes back to normality on Sep 21st when the hive is replaced with a fresh healthy colony.

In the active season, between late April and September, the results (Fig. 9) show that the frame monitored for vibrations in Colony No 3 did not experience significantly higher vibrations than the rest of the apiary, but that they consistently responded with substantially greater buzzing response than any of the other colonies during their difficult times, and that this signal immediately returned to normality upon replacing the colony with a fresh one.

## Discussion

A quantitative implementation of a simple experiment has yielded an unexpected plethora of exciting new science. Using an artificial short vibrational pulse delivered on a colony at random times, provides a short-lived drop of colony-induced vibrational signal, followed by a relatively long-lived positive buzzing response, sometimes accompanied with individual whooping signals. All three outcomes appear to be independent from each other. The measurement promises to provide, non-invasively, colony overall mobility, the clustering of the colony, and its ‘restfulness’. As far as we know there is no reflex arc described in invertebrates, their nervous system is quite different to that of vertebrates, and our measurement is sensing the simultaneous response of hundreds or thousands of individuals as opposed to one, in the usual meaning of the reflex arc. However, the phenomenon we investigated (i) is a rapid response to an external stimulus, (ii) is a reaction to a signal that can be interpreted as ‘danger/dangerous’, (iii) provides vibrations as an outcome, necessarily involving motor-neurons, (iv) decreases in strength with habituation^4^, and (v) is shown to vary with time and physiological status.

The positive, long-lived buzzing response is only explored within an ethological, phenomenological approach. In healthy colonies, it seems to be nearly absent in the active season, with occasional appearance early in the morning. It becomes very strong with the winter, then vanishes again in the late spring. Not every colony exhibits a strong response in the winter (4 out of 9), and this does not seem to be due to our poor choice in pulse central frequency, or poor transmission of the vibration to the colony, except for one (colony 2). We propose that perhaps the strength of this signal reflects the ‘restfulness’ of the colony, as it is mostly seen in the winter and in the early morning, and honeybees during the active season can be very busy until late in the night, working on the large amount of resources that have been collected during the day.

One colony out of nine developed serious health deterioration during the active season, and it is the only one exhibiting a strong, easily measurable positive long-lived buzzing response, consistently throughout the summer whilst the colony was slowly deteriorating. Although any generalisation is presently impossible, this gives a tantalising hope for this signal to be an indicator of at least some health disorders, at least in the active season.

Critical listening of the whooping signals stimulated by the artificial pulse suggests that these come from a few isolated individuals, are a poor representation of the colony, and were therefore not further investigated in this study. However, using a simple unsupervised PCA analysis on the collection of response measurements, we managed to discriminate well the whooping signals from the long lived buzzing response. In future work where perhaps a stronger stimulus is used, the occurrences of stimulated whooping signals and their statistics might provide a new independent response to explore.

There are numerous works on sound and vibration signals that have been shown to be communication cues in honeybee colonies. The artificial pulse that we have used coincidentally matches quite well the honeybee ‘whooping signal’^4^, which could lead to the conclusion that we have (unintentionally) modulated the physiological status of the colony, assuming that it was a communication signal for honeybees. This, however, is most unlikely, because the signal we have used (i) is very short compared with common communication signals and (ii) stimulates the strongest responses during non-active periods of the colony.

The honeybee immobilisation phenomenon that is explored here is highly repeatable and can be assessed multiple times within one minute, as demonstrated in our experiments. It does not seem to be part of any potential reflex arc phenomenon, in particular it does not seem to fade away with habituation. This further supports our claim that it is independent from the positive response that we are also detecting, and yields an unexpected meaningful additional information regarding the colony’s status, i.e. its overall mobility. We suspect that the higher the colony’s mobility is, the higher the vibrational signal is before the pulse, and the greater the signal loss is, immediately after the pulse.

The accelerometer signal drop following the application of the stimulus (Fig. 3) seems well correlated with the visually assessed immobilisation of the bees in the colony (Fig. 4) but the time courses are quite different, in particular the accelerometer signal drop seems much shorter. However, we have not carefully looked into how honeybee colony overall mobility affects the accelerometer signal, and it is also possible that visually assessing the pulse’s ability to immobilise the bees might provide different exponential recovery functions at different times of the days, or different seasons, and that the absolute number of bees residing on the frame will contribute to the accelerometer signal change. Substantial further work is required to explore these interesting questions. Eventually, when the accelerometer signal drop is calibrated against the immobilisation of the bees, it will be possible to assess whether the freezing phenomenon varies with time, if at all. In our visually assessed experiments we have always been able to freeze the bees with the same ability, but in the future, more careful experimentation might show mild variations of the phenomenon during the year or during the day.

Our study, so far, extensively used a short pulse centred on 340 Hz, which unfortunately is not optimal for immobilisation purposes, because such frequency does not make best use of our transducer’s frequency response (Figs. 5, 8). It is possible and likely that a frequency around 500 Hz will provide better and more consistent transmission of a stronger vibrational signal to the bees, and therefore better immobilisation, but it remains to be seen whether this will affect the positive buzzing response or not. We cannot, in the present study, showcase meaningful measurements of the immobilisation-induced, short-lived drop of signal, but we provide strong evidence showing that this will very soon be possible.

The drop of accelerometer signal due to the immobilisation is weak and impossible to hear by critical listening, but it is measurable, and we have means to enhance it (e.g. by providing a stronger vibration, e.g. centred around 500 Hz). It will however probably remain difficult to measure it with a microphone, as bees moving on a frame provide a strong accelerometer signal but not much sound, if at all (microphone measurements have otherwise yielded numerous exciting scientific discoveries in honeybee science^11^). On the other hand, the positive, long-lived buzzing response that follows our artificial pulse, is likely to be equally well picked up by an appropriately placed microphone in the hive. Such microphone measurements will not be affected by the honeycomb density changes, which might provide an enhanced consistency in the results.

The signal drop revealing the bees mobility was not deemed reliable enough, at this stage, for careful interpretation. We seem however to have reached the threshold required to stimulate the bees most of the time, and the data from the pilot study in Fig. 8B demonstrates a considerable enhancement of the buzzing response in the winter, from November to early April, a mild decrease in the middle of the day, and some relatively strong responses in the early morning during the active season. Even at times where the driven vibration is very strong, the bees do not seem to react to the stimulus when they are very active, in the summer and in the evening. The occasional early morning responses during the active season are weaker than the winter ones, perhaps because they do not take place when the clustering has already formed, but are unlikely to originate from the honeycomb density variation, because strong winter reactions are recorded immediately at the start of the winter, at a time when the honeycomb density is still very high.

In the winter, it is inevitable that the positive buzzing reaction that follows the stimulus will increase, sometimes for up to four of five seconds, the metabolic rate of the colony, and their energy consumption, perhaps somewhat weakening the claim that our measurement (as for any established reflex arc measurement) is truly ‘non-invasive’. However, in the wild, a colony living in a tree will experience similar, if not much stronger, vibrational signals originating from branches colliding with other trees, woodpeckers, etc. Furthermore, our pulse strength was set to the minimum allowing, occasionally, the detection of the bees’ reaction. Finally, an hourly increased metabolic rate for five seconds only represents, at worse, a metabolic percentage enhancement of 0.14%, and in a practical beekeeping implementation of this method, there is probably no need to run a stimulation experiment every hour.

The large daily variations in the magnitude of the vibration that reaches the bees are so large and so rapid, that they are most probably due to changes in the instantaneous number of bees residing on the frame. Exciting further work is required to explore whether one might be able to calibrate the effect, perhaps by measuring the full vibrational modes of the frame, in order to estimate the number of bees residing on any frame. The presence of the bees will also change the temperature of the frame and this will also need to be calibrated.

The electromagnetic shaker that we have used probably has a very poor frequency response, as shown in Fig. 5, but it is inexpensive, effective, used at one frequency only for the critical experiments, small enough to be housed conveniently in a weatherproof box, and works very well over more than one year of use. It promises the possibility of implementing this experiment with remarkably simple and inexpensive hardware.

When our method is improved to bring successful non-invasive measurements of the colony’s overall mobility, additional valuable information will be discernible, including indirect measurements of the colony’s metabolic rate, winter cluster formation, health-disorder induced decreased mobility, etc.

## Methods

The recording of the accelerometer signals was done under the Ubuntu O.S. with home-built software that has been described elsewhere^9,10^, a sound card Alesis iO4 (Cumberland, USA) for the pilot study, and a sound card M-Track 8 (M-Audio, USA) for the outdoors hive study. The transmission of the stimuli was undertaken with home-built software combining bash and Octave (GNU) codes, using stimuli driven every 60 min + /− a randomised time with standard deviation of 15 min. Data analysis was undertaken with a collection of home-built code in Octave (GNU). Video analysis was conducted with matlab® code on full HD footage acquired at 50 FPS (Sony 4K FDR-AX100E handycam, China). The outcome of the honeybee colony inspections included qualitative and quantitative assessments, and was were logged consistently with the help of the BEEP software (beep.nl, Driebergen-Rijsenburg, NL). Colony No. 3 was deemed to be queenless through visual inspection from April 13^th^ onwards, where the colony was observed to be broodless with relatively low stores of uncapped honey and the frames were in very poor condition. Attempts were made subsequently, during the nectar flow season, to requeen this colony by (i) giving queen cells, then (ii) providing a mobile queen and (iii) frames containing fresh brood from other colonies. All attempts failed until a mobile queen was introduced on the 20th of May from a neighbouring colony (No. 6). The queen was accepted by the colony, and capped brood and capped honey could be seen in the hive in mid-June. An inspection on July 5th revealed that the colony had lost their queen again, some capped brood was remaining and the colony had low honey stores. Subsequently, no laying took place in July and in the following inspection on August 7th the colony was in a very poor state with hardly any worker bees left, no honey, and no brood. The colony was eventually replaced by a completely new colony from a nearby apiary on the 21st of September, with plenty of worker bees, brood, honey, and an active queen.

### Supplementary Information

Supplementary Legends.

Supplementary Figures.

Supplementary Information 2.

Supplementary Information 2.

Supplementary Video 1.

Supplementary Video 2.

## Data Availability

All relevant data are in the paper and its supplementary material. The raw accelerometer responses are supplied as flac files, with the time of the measurement given as Unix epoch in the file name. All relevant Matlab^®^, Octave and Bash code can be found on GitHub: https://github.com/sci3bencsm/Honeybee_reflex_arc.

## References

[1] Walker HK, Hall WD, Hurst JW, Walker HK (1990). The plantar Reflex. Clinical Methods: The History, Physical, and Laboratory Examinations.

[2] Futagi Y, Toribe Y, Suzuki Y (2012). The grasp reflex and moro reflex in infants: Hierarchy of primitive reflex responses. Int. J. Pediatr..

[3] Woods EF (1959). Electronic prediction of swarming in bees. Nature.

[4] Ramsey M, Bencsik M, Newton MI (2012). Long-term trends in the honeybee ‘whooping signal’ revealed by automated detection. PLOS ONE.

[5] Frings H, Little F (1957). Reactions of honey bees in the hive to simple sounds. Science.

[6] Spangler HG (1969). Suppression of honey bee flight activity with substrate vibration. J. Econ. Entomol..

[7] Stefanec M, Oberreiter H, Becher MA, Haase G, Schmickl T (2021). Effects of sinusoidal vibrations on the motion response of honeybees. Front. Phys..

[8] Michelsen A, Kirchner WH, Lindauer M (1986). Sound and vibrational signals in the dance language of the honeybee, Apis mellifera. Behav. Ecol. Sociobiol..

[9] Ramsey M (2020). The prediction of swarming in honeybee colonies using vibrational spectra. Sci. Rep..

[10] Ramsey M, Bencsik M, Newton MI (2018). Extensive vibrational characterisation and long-term monitoring of honeybee dorso-ventral abdominal vibration signals. Sci. Rep..

[11] Uthoff C, Homsi MN, von Bergen M (2023). Acoustic and vibration monitoring of honeybee colonies for beekeeping-relevant aspects of presence of queen bee and swarming. Comput. Electron. Agric..

