# Supplementary material for: Quantitative assessments of honeybee colony’s response to an artificial vibrational pulse resulting in non-invasive measurements of colony’s overall mobility and restfulness
Source: Sci Rep. 2024 Feb 15;14:3827. doi: 10.1038/s41598-024-54107-8 (PMC10869359; doi:10.1038/s41598-024-54107-8)
Supplement: Supplementary file 1 — Supplementary Legends. [file 41598_2024_54107_MOESM1_ESM.docx]

**Supplementary Video 1**

A video demonstrating the outcome of processing the honeybee immobilisation experiment. Top left: raw video footage of the bees in the observation hive. To right: coarsened-resolution video of the difference image between two consecutive (grey scaled) images of the raw video. The colour coding has been altered to improve the visualisation of the very brief darkening of the outcome, which reveals the immobilisation of the bees. Bottom graphs: the green curve represents the average pixel intensity of the difference image shown on the top right insert. Any decrease in honeybee overall mobility is reflected by a decrease in this curve. The blue and orange curves represent the envelope of the magnitude of the acceleration measured in the honeycomb, averaged over the time duration of one frame of the video (20ms). The graph allows careful, semi-quantitative comparison of the timings of the application of the artificial vibration, and the resulting honeybee immobilisation.

The soundtrack of the video is originating from the accelerometers in the frame. Said sensors are not visible as they have been embedded in the honeycomb by the bees themselves. They are 7 cm apart, equally spaced around the centre of the honeycomb, on a horizontal line.

**Supplementary Video 2**

A video demonstrating the outcome of the PCA analysis on the honeybee reactions to an artificial stimulus. A collection of fifty recordings is showcased, stacked in rapid succession. In reality, each experiment is undertaken approximately every hour. The experiments are not sorted in chronological order, they are shown from the strongest to weakest one. Top graph: envelope of the accelerometer signal, smoothed with a moving average with window of 4.5 ms. The data shows one second of data before the artificial stimulus, the artificial stimulus, and four seconds of data after its application. Bottom graph: outcome of the PCA reconstruction using a limited number of PC scores, for the section of the envelope that is immediately after the artificial pulse. The blue curve shows the outcome of using PC scores 1 and 2, only, and provides an excellent description of the overall colony buzzing response. The orange curve provides the reconstructed wave form when using PC scores 1 to 15, highlighting the envelope changes due to a few isolated individuals in the vicinity of the accelerometer.

The soundtrack of the video is originating from the accelerometers in the frame, and allows, by critical listening, subjective validation of the claim that we have successfully discriminated the overall buzzing response of the colony from the less reliable occasional whooping signals coming from isolated individuals in the vicinity of the sensor.
