# Supplementary material for: Quantitative assessments of honeybee colony’s response to an artificial vibrational pulse resulting in non-invasive measurements of colony’s overall mobility and restfulness
Source: Sci Rep. 2024 Feb 15;14:3827. doi: 10.1038/s41598-024-54107-8 (PMC10869359; doi:10.1038/s41598-024-54107-8)
Supplement: Supplementary file 2 — Supplementary Figures. [file 41598_2024_54107_MOESM2_ESM.docx]

**Supplementary Graphs**

A set of eight graphs showcasing the outcome of the experiment on the 8 hives kept outdoors. The figure caption for the top and bottom graphs are the same as those supplied for Figure 8 A and B, with the data originating here from the apiary with the 8 hives being kept outdoors.


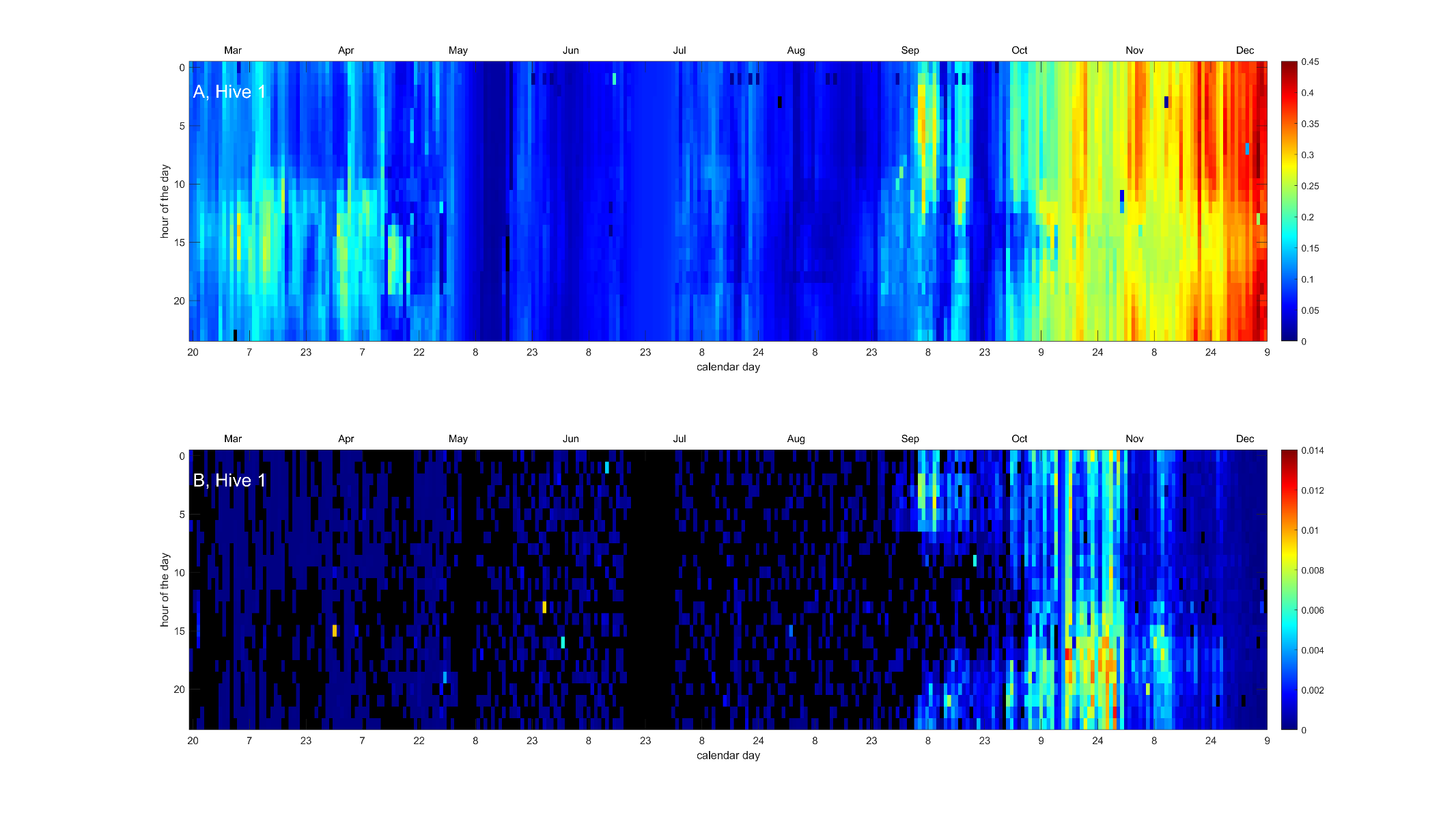


**A – Variation of the magnitude of the vibration reaching the honeycomb on which the bees response is measured, for Hive No. 1. The vertical axis is the time of the day, the horizontal axis the day of the year, data has been interpolated in order to allow hourly visualisation (in reality pulses are driven at randomised times, approximately hourly) and pixel intensity reflects the magnitude of the vibration on a linear scale, in arbitrary units. B – Variation of the magnitude of the positive response of the bees within the four seconds that follow the artificial pulse, with the same formatting as seen in -A-. The pixel intensity reflects the subtraction of the mean vibration recorded after the pulse, from the mean vibration recorded one second before the pulse.**


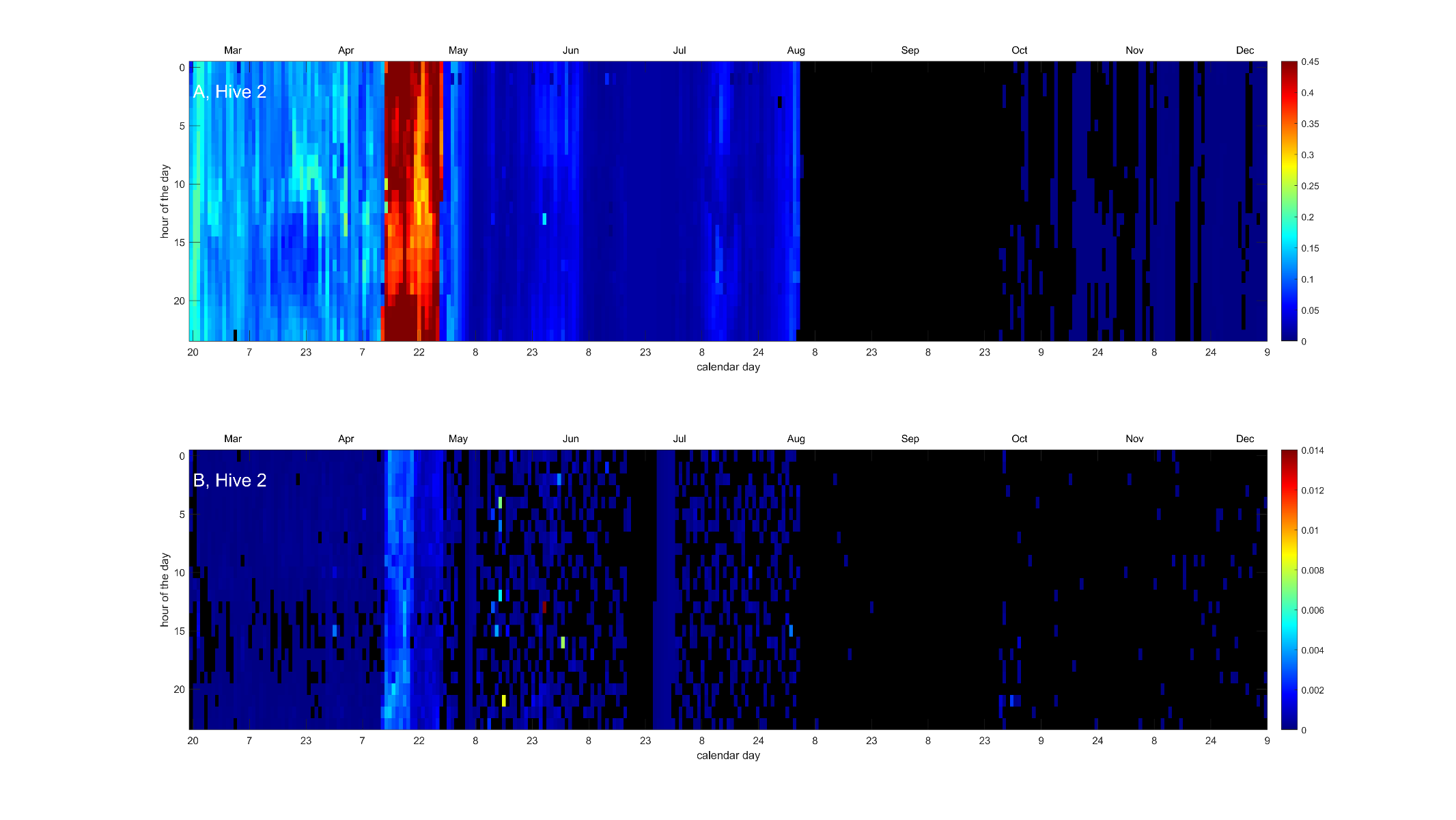


**A – Variation of the magnitude of the vibration reaching the honeycomb on which the bees response is measured, for Hive No. 2. The vertical axis is the time of the day, the horizontal axis the day of the year, data has been interpolated in order to allow hourly visualisation (in reality pulses are driven at randomised times, approximately hourly) and pixel intensity reflects the magnitude of the vibration on a linear scale, in arbitrary units. B – Variation of the magnitude of the positive response of the bees within the four seconds that follow the artificial pulse, with the same formatting as seen in -A-. The pixel intensity reflects the subtraction of the mean vibration recorded after the pulse, from the mean vibration recorded one second before the pulse.**


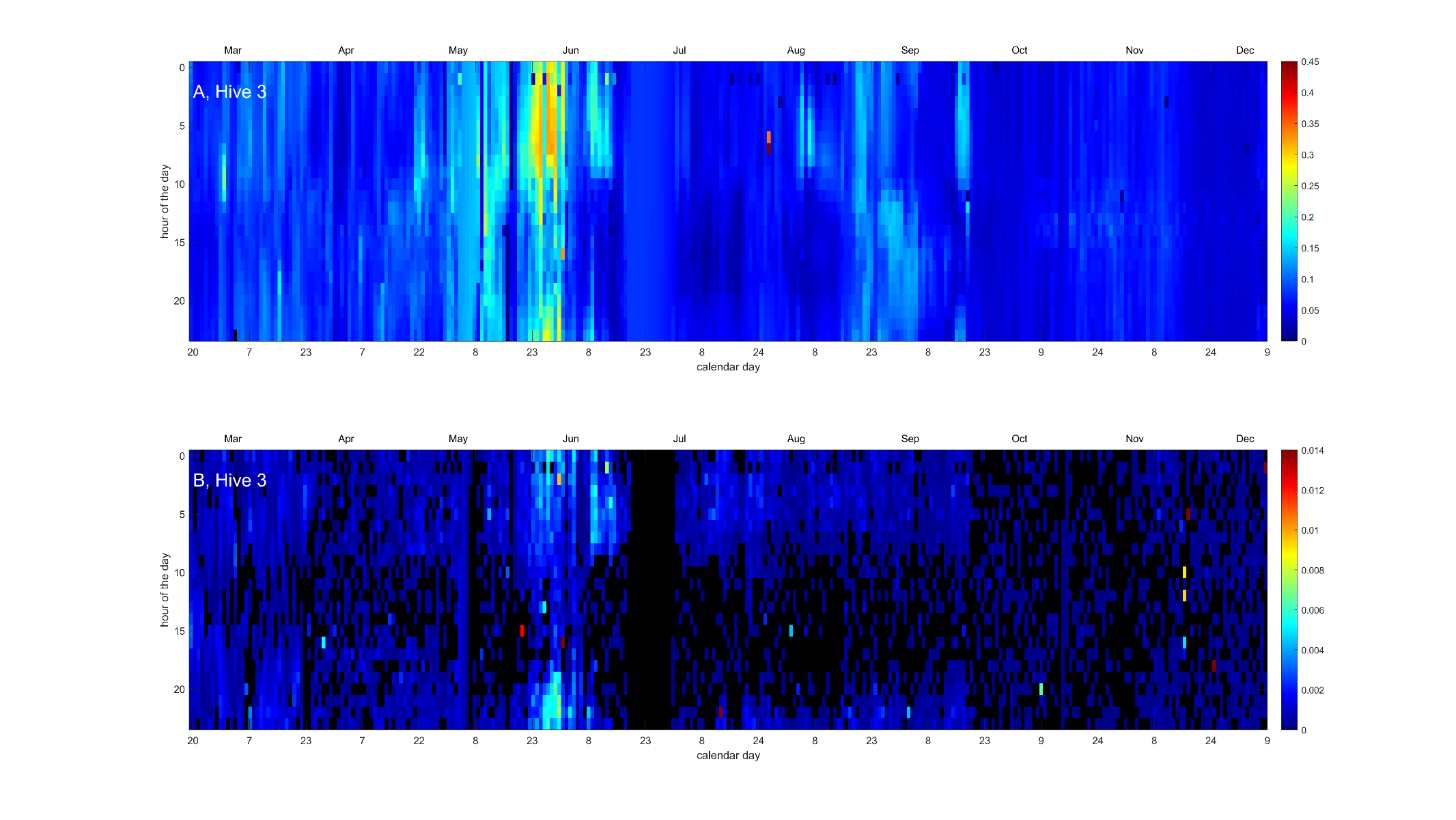


**A – Variation of the magnitude of the vibration reaching the honeycomb on which the bees response is measured, for Hive No. 3. The vertical axis is the time of the day, the horizontal axis the day of the year, data has been interpolated in order to allow hourly visualisation (in reality pulses are driven at randomised times, approximately hourly) and pixel intensity reflects the magnitude of the vibration on a linear scale, in arbitrary units. B – Variation of the magnitude of the positive response of the bees within the four seconds that follow the artificial pulse, with the same formatting as seen in -A-. The pixel intensity reflects the subtraction of the mean vibration recorded after the pulse, from the mean vibration recorded one second before the pulse.**


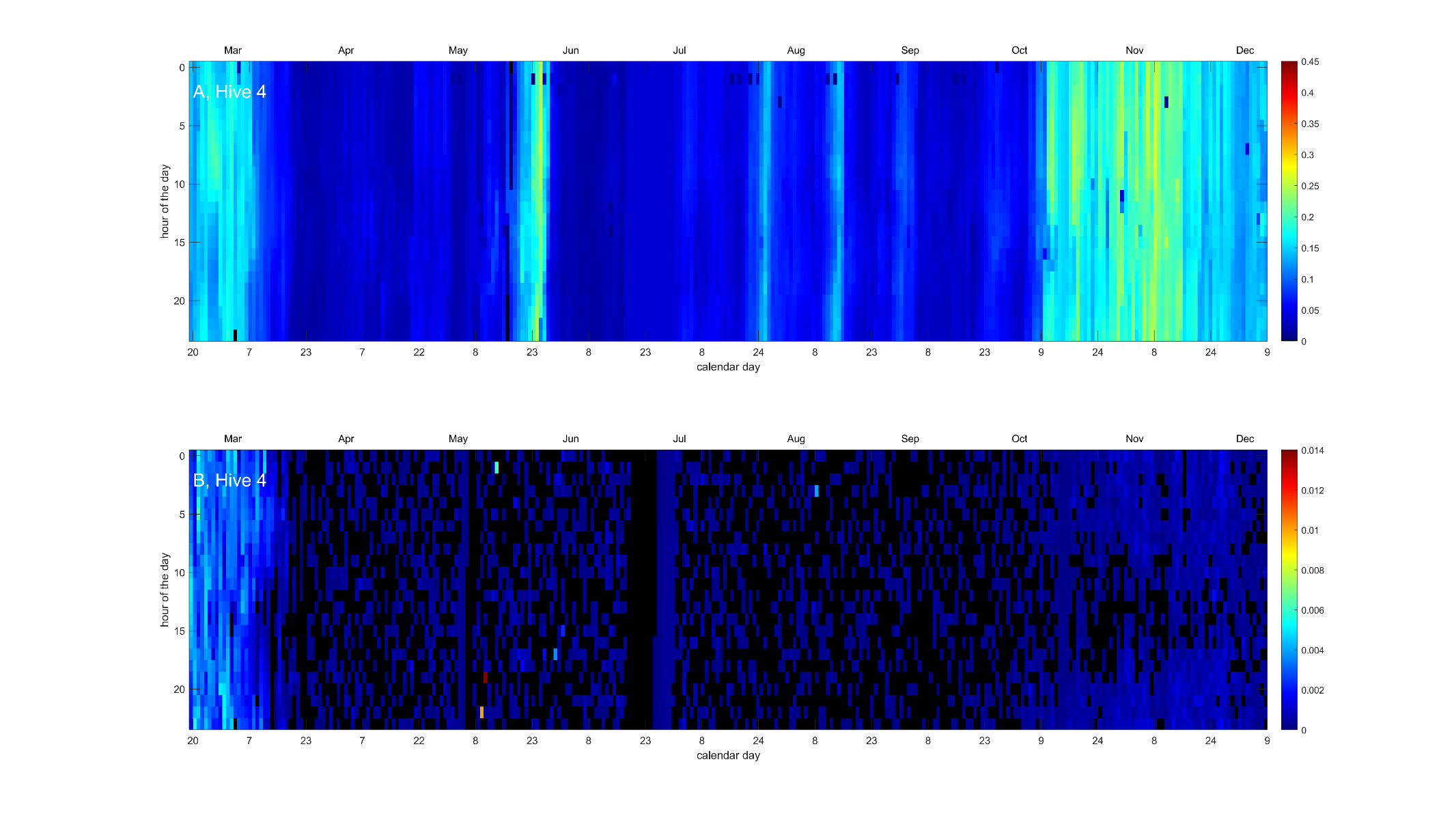


**A – Variation of the magnitude of the vibration reaching the honeycomb on which the bees response is measured, for Hive No. 4. The vertical axis is the time of the day, the horizontal axis the day of the year, data has been interpolated in order to allow hourly visualisation (in reality pulses are driven at randomised times, approximately hourly) and pixel intensity reflects the magnitude of the vibration on a linear scale, in arbitrary units. B – Variation of the magnitude of the positive response of the bees within the four seconds that follow the artificial pulse, with the same formatting as seen in -A-. The pixel intensity reflects the subtraction of the mean vibration recorded after the pulse, from the mean vibration recorded one second before the pulse.**


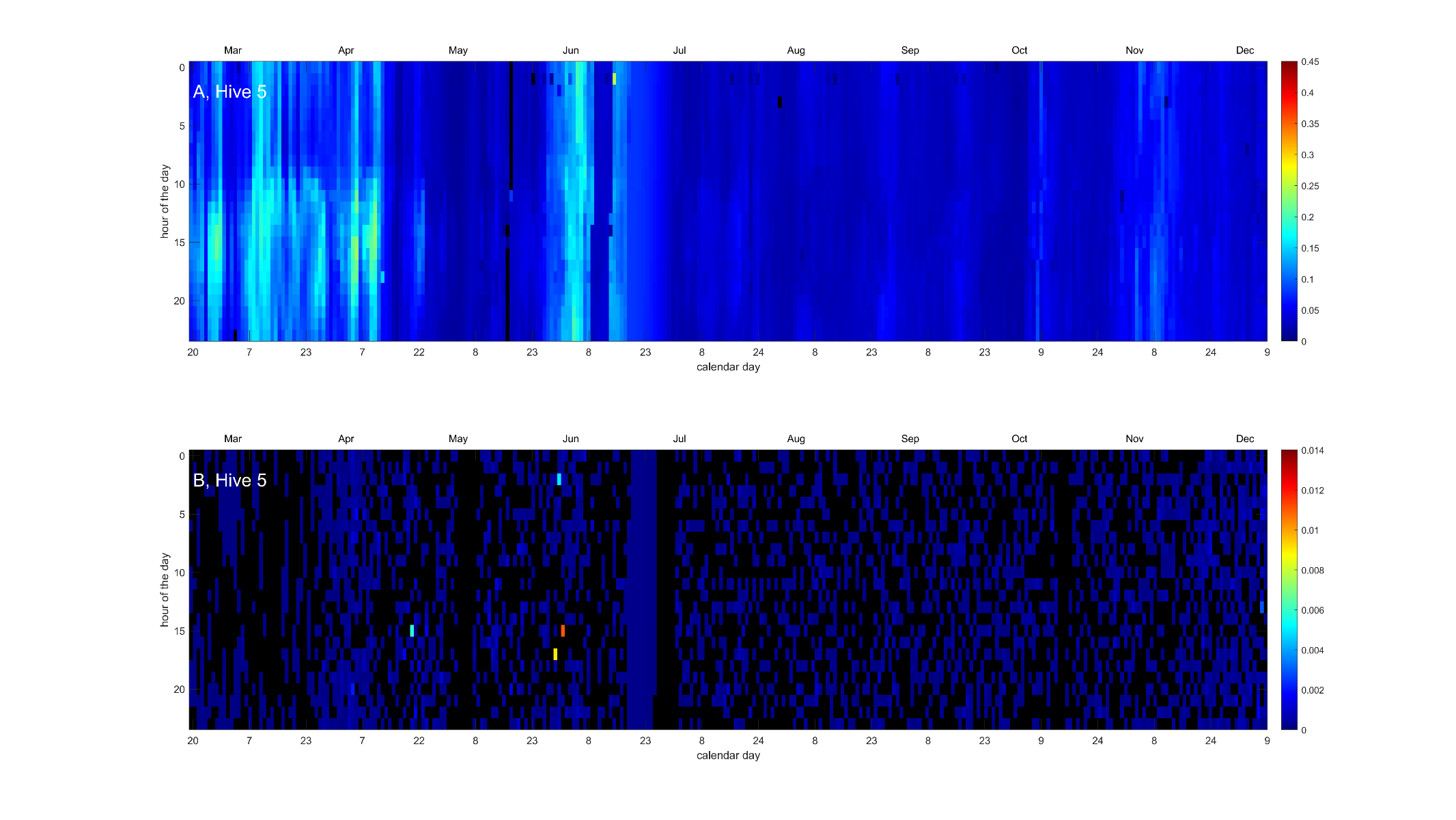


**A – Variation of the magnitude of the vibration reaching the honeycomb on which the bees response is measured, for Hive No. 5. The vertical axis is the time of the day, the horizontal axis the day of the year, data has been interpolated in order to allow hourly visualisation (in reality pulses are driven at randomised times, approximately hourly) and pixel intensity reflects the magnitude of the vibration on a linear scale, in arbitrary units. B – Variation of the magnitude of the positive response of the bees within the four seconds that follow the artificial pulse, with the same formatting as seen in -A-. The pixel intensity reflects the subtraction of the mean vibration recorded after the pulse, from the mean vibration recorded one second before the pulse.**


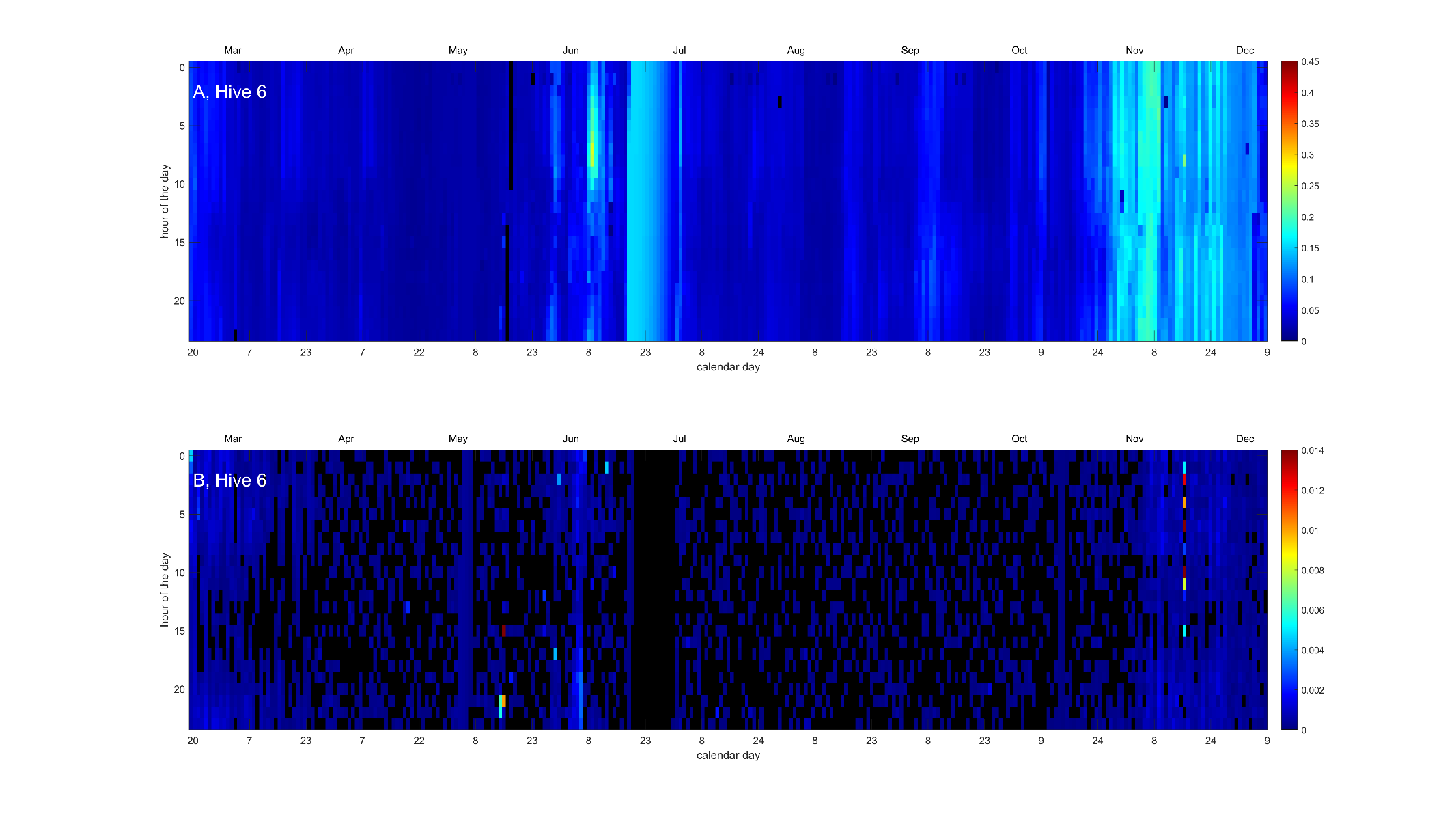


**A – Variation of the magnitude of the vibration reaching the honeycomb on which the bees response is measured, for Hive No. 6. The vertical axis is the time of the day, the horizontal axis the day of the year, data has been interpolated in order to allow hourly visualisation (in reality pulses are driven at randomised times, approximately hourly) and pixel intensity reflects the magnitude of the vibration on a linear scale, in arbitrary units. B – Variation of the magnitude of the positive response of the bees within the four seconds that follow the artificial pulse, with the same formatting as seen in -A-. The pixel intensity reflects the subtraction of the mean vibration recorded after the pulse, from the mean vibration recorded one second before the pulse.**


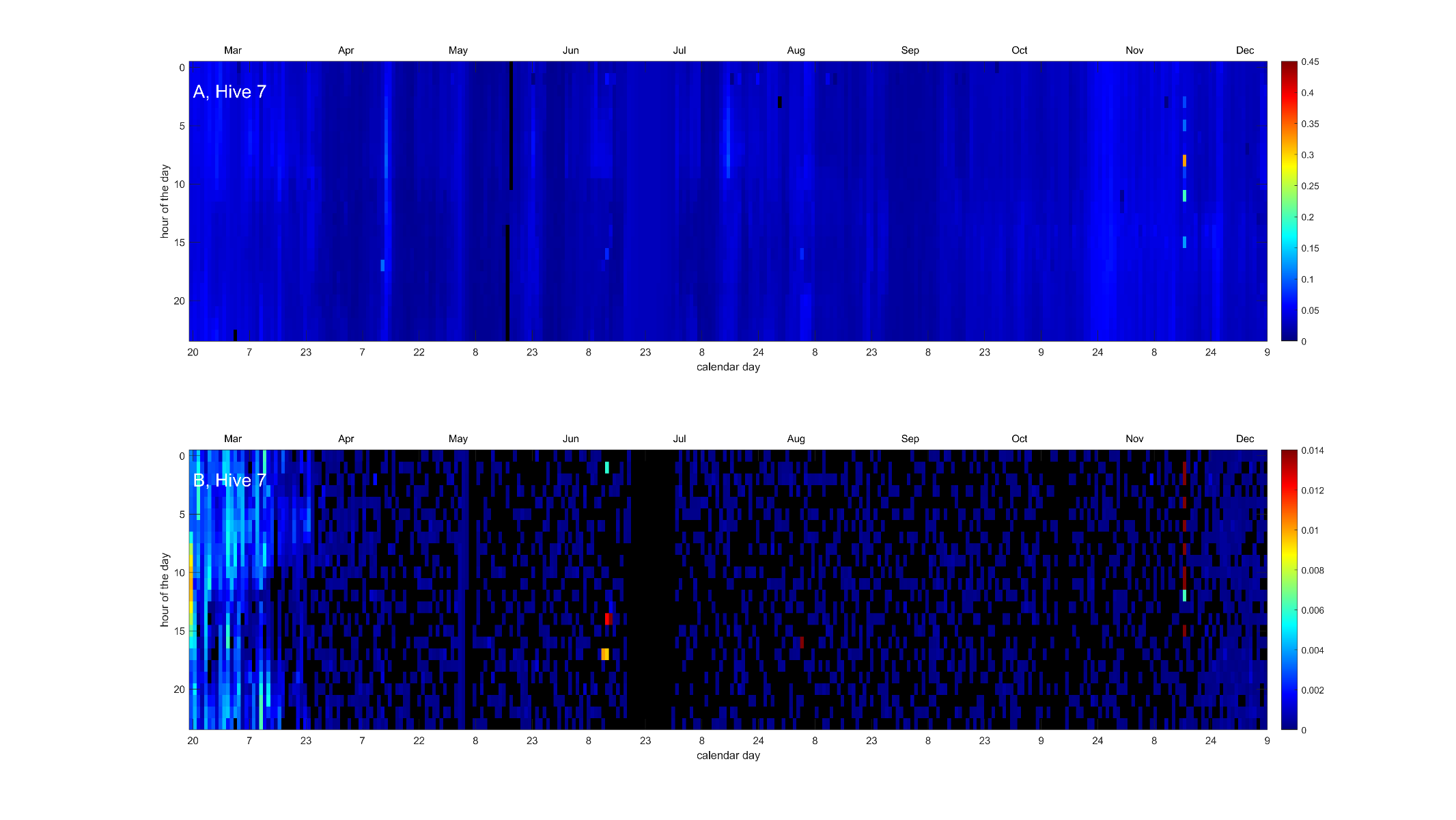


**A – Variation of the magnitude of the vibration reaching the honeycomb on which the bees response is measured, for Hive No. 7. The vertical axis is the time of the day, the horizontal axis the day of the year, data has been interpolated in order to allow hourly visualisation (in reality pulses are driven at randomised times, approximately hourly) and pixel intensity reflects the magnitude of the vibration on a linear scale, in arbitrary units. B – Variation of the magnitude of the positive response of the bees within the four seconds that follow the artificial pulse, with the same formatting as seen in -A-. The pixel intensity reflects the subtraction of the mean vibration recorded after the pulse, from the mean vibration recorded one second before the pulse.**


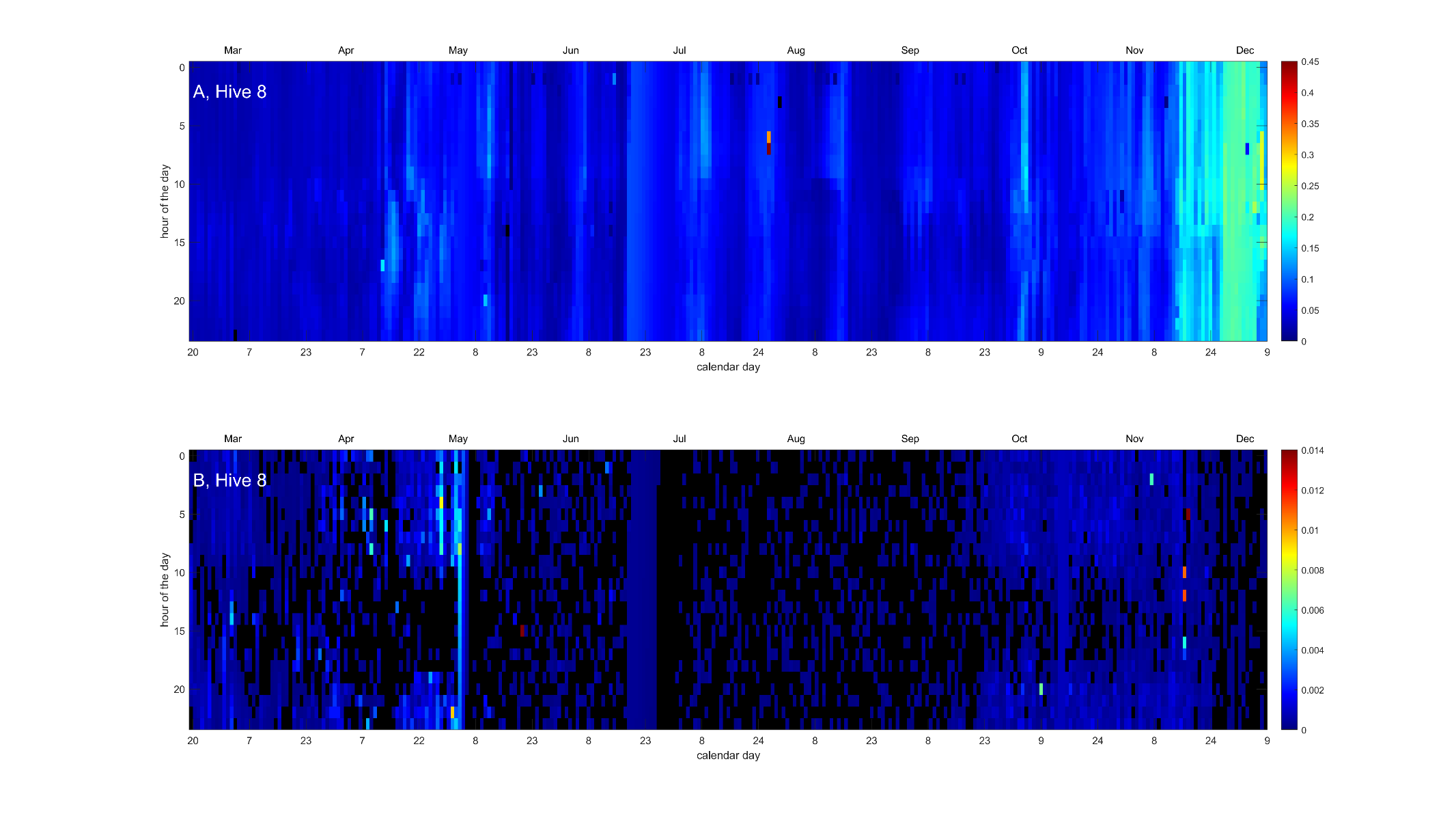


**A – Variation of the magnitude of the vibration reaching the honeycomb on which the bees response is measured, for Hive No. 8. The vertical axis is the time of the day, the horizontal axis the day of the year, data has been interpolated in order to allow hourly visualisation (in reality pulses are driven at randomised times, approximately hourly) and pixel intensity reflects the magnitude of the vibration on a linear scale, in arbitrary units. B – Variation of the magnitude of the positive response of the bees within the four seconds that follow the artificial pulse, with the same formatting as seen in -A-. The pixel intensity reflects the subtraction of the mean vibration recorded after the pulse, from the mean vibration recorded one second before the pulse.**
